# Health care-seeking behavior for childhood illnesses in western Kenya: Qualitative findings from the Child Health and Mortality Prevention Surveillance (CHAMPS) Study

**DOI:** 10.12688/gatesopenres.14866.3

**Published:** 2024-09-27

**Authors:** Sarah Ngere, Maria Maixenchs, Sammy Khagayi, Peter Otieno, Kennedy Ochola, Kelvin Akoth, Aggrey Igunza, Benard Ochieng, Dickens Onyango, Victor Akelo, John Blevins, Beth A. Tippett Barr

**Affiliations:** 1 Kenya Medical Research Institute-Center for Global Health Research, Kisumu, Kenya; 2 ISGlobal, Hospital Clínic, Universitat de Barcelona, Barcelona, Spain; 3 Kisumu County Department of Health, Kisumu, Kenya; 4 U.S. Centers for Disease Control and Prevention, Kisumu, Kenya; 5 Emory University, Atlanta, Georgia, USA; 6 Nyanja Health Research Institute, Salima, Malawi

**Keywords:** Health-seeking behavior, childhood illness, under-5 mortality, qualitative research, traditional medicine

## Abstract

**Background:**

Child mortality in Kenya is 41 per 1,000 live births, despite extensive investment in maternal, newborn, and child health interventions. Caregivers’ health-seeking for childhood illness is an important determinant of child survival, and delayed healthcare is associated with high child mortality. We explore determinants of health-seeking decisions for childhood illnesses among caregivers in western Kenya.

**Methods:**

We conducted a qualitative study of 88 community members between April 2017 and February 2018 using purposive sampling in an informal urban settlement in Kisumu County, and in rural Siaya County. Key informant interviews, semi-structured interviews and focus group discussions were performed. We adopted the Partners for Applied Social Sciences model focusing on factors that influence the decision-making process to seek healthcare for sick infants and children. The discussions were audio-recorded and transcribed. Data management was completed on *Nvivo®* software. Iterative analysis process was utilized and themes were identified and collated.

**Results:**

Our findings reveal four thematic areas: Illness interpretation, the role of social relationship on illness recognition and response, medical pluralism and healthcare access. Participants reported some illnesses are caused by supernatural powers and some by biological factors, and that the illness etiology would determine the health-seeking pathway. It was common to seek consensus from respected community members on the diagnosis and therefore presumed cause and necessary treatment for a child’s illness. Medical pluralism was commonly practiced and caregivers would alternate between biomedicine and traditional medicine. Accessibility of healthcare may determine the health seeking pathway. Caregivers unable to afford biomedical care may choose traditional medicine as a cheaper alternative.

**Conclusion:**

Health seeking behavior was driven by illness interpretation, financial cost associated with healthcare and advice from extended family and community. These findings enrich the perspectives of health education programs to develop health messages that address factors that hinder prompt health care seeking.

## Background

Globally, under-five mortality rates (U5MR) have declined; however sub-Saharan Africa on average has 74 deaths per 1,000 live births, compared to the global rate of 38 deaths per 1,000 live births in 2020 ^1^. In Kenya, U5MR has reduced from 101 to 41 per 1000 live births since 1990 ^2^, but achieving the 2030 Sustainable Development Goal of 25 deaths per 1,000 live ^3, 4^, will require a deeper understanding of how decision-making is made by the family before formal healthcare is sought. A large proportion of children still die due to delays in seeking appropriate care attributed by caregivers choosing to first use over-the-counter (OTC) medication and/or traditional medicine ^5– 7^.

Health seeking behavior is defined as a complex process guided by a decision-making process that is governed by socio-cultural, structural and economic factors ^7– 11^. Belief systems ingrained by cultural beliefs define causes of illness under two broad categories: Those caused by supernatural etiology and those caused by biological pathogens. The category determined to cause an illness provides the basis for health-care seeking decision making ^12, 13^. Studies conducted in Tanzania, report that the determination of a natural or supernatural cause of illness is made by the caregiver’s perception of severity and etiology ^14, 15^. Previous studies indicate that lack of knowledge and delay in recognition of the severity of an illness are reasons for delayed health care seeking ^16, 17^. Lack of awareness of severity informs health care decisions that are not favorable to the child’s health such as use of OTC medications and engagement of traditional healers rather than formal health care ^17– 19^. 

Community based information is needed regarding drivers to health care decision making. Understanding what governs caregivers’ health seeking decisions for childhood illnesses is essential to understanding the health seeking behavior. This is important in formulating policies and strategies that optimize response to illness. Using the constructs of the Partners for Applied Social Sciences (PASS) model, we explored how cultural, social and economic factors drove health care decision making.

## Methods

### Ethics

This study was part of Child Health and Mortality Surveillance (CHAMPS) Kenya study which was approved by the KEMRI Ethics Review Committee (KEMRI Protocol # 3313). Each participant provided verbal informed consent. We used codes to name audio recordings, stored them in an offline device, and deleted them from the audio recorders. All study staff involved in data collection and transcription were trained in the handling of confidential information. All laptops with data were password-protected; the storage device was kept in lockable cabinet and was only accessed by the authorized staff. No personal identifiers were used in reporting or publication.

### Study setting and design

For this analysis, we collected data among community members of Manyatta (urban informal settlement) and Karemo (rural) Health and Demographic Surveillance System (HDSS) sites located in Kisumu and Siaya counties respectively between April 2017 and February 2018. A descriptive cross-sectional study was conducted during the formative phase of the Socio-Behavioral Science (SBS) study of Child Health and Mortality Prevention (CHAMPS) Network; these methods have been published elsewhere ^20– 22^. The SBS aim was to evaluate the feasibility (i.e. acceptability, practicality and implementation) and ethical considerations of child mortality surveillance. We employed a qualitative design using a combination of ethnography and phenomenological approaches. The data collection methods involved for the current analysis included key informant in-depth interviews (KIIs), focus group discussions (FGDs) and semi-structured interviews (SSIs). Interview guides were developed in English and translated into Swahili and Dholuo. The multi-approach method was used to triangulate findings across all data sources.

### Study participants

Participants were purposively selected from a predetermined sampling frame outlined in the CHAMPS socio-behavioral protocol ^20^. Participant categories included: Community representatives and religious leaders (Christian and Muslim representatives); community leaders (opinion leaders, chiefs, assistant chiefs and village elders) and other community members; Healthcare providers in the formal healthcare system, traditional birth attendant midwives (TBA); and traditional healers.

### Theoretical framework

The study drew on the Partners for Applied Social Sciences (PASS) model – developed within the PASS International organization to explore contextual factors that drive health care seeking decisions. The PASS model has four categories which independently or interdependently determine the health care choice to use biomedical, traditional medicine or a combination of both. These include: i) illness perception; ii) social values and stigma; iii) social pressure and support; iv) access to care and resource seeking ^12^. The availability of health care resources within an area, accessibility to these resources by the population, and accommodation between the health services and people’s needs are the basic determinants for access to healthcare and ultimately healthcare behavior. From the perspective of the PASS model, illness is not only an individual matter but a social matter where health care decisions can be determined by the community.

### Data management and analysis

All interviews and focus group discussions were audio recorded, transcribed verbatim, and then translated from Dholuo or Swahili to English. A code book was developed and an iterative analysis process was performed. *Nvivo®* software was used to organize and manage data and code themes from transcribed discussion. We applied deductive coding based on constructs of the PASS framework. Then we assigned codes to segments of the transcribed texts and searched for themes from the coded texts. The lead author interpreted the themes and summarized them. Quotes were selected based on their clear representation of themes.

### Ethical considerations

This study was part of Child Health and Mortality Surveillance (CHAMPS) Kenya study which was approved by the KEMRI Ethics Review Committee (KEMRI Protocol # 3313). Each participant provided a verbal informed consent. We used codes to name audio recordings, stored them in an offline device, and deleted them from the audio recorders. All study staff involved in data collection and transcription were trained in the handling of confidential information. All laptops with data were password-protected; the storage device was kept in a lockable cabinet and was only accessed by the authorized staff. No personal identifiers were used in reporting or publication.

## Results

A total of 88 participants were interviewed in 29 IDIs, 5 FGDs and 11 SSIs, of whom fifty-one (58%) were female. Thirty-eight (43%) had at least secondary education or higher, and most 80/88 (91%) were Christian ( Table 1).

**Table 1.** Socio-demographic characteristics of study participants.

	Caregivers	Health workers	Religious leaders	TBA	Traditional healer	Community leaders	Total
**Gender**							
Female	33	9	1	3	1	4	51(57.9%)
Male	20	4	6	0	0	7	37(42%)
**Age**							
<30	26	3	0	0	1	0	30(34%)
31–49	19	8	3	0	0	4	34(38.6%)
50+	8	2	4	3	0	7	24(27%)
**Highest education**							
None	1	0	0	0	0	0	1(0.0%)
Upper Primary	18	0	5	1	0	4	28(31.8%)
Lower Primary	5	0	0	1	0	0	6(6.8%)
Some Secondary	14	0	1	0	0	0	15(17%)
Secondary	7	0	0	1	1	3	12(13.6%)
College and above	8	13	1	0	0	4	26(29.6)
**Source of income**							
Formal employment	1	13	4	0	0	3	21(23.9%)
Self-employment	32	0	3	3	1	4	43(48.9%)
Other	20	0	0	0	0	4	24(27.2%)
**Religion**							
Christian	48	12	6	3	1	10	80(90.9%)
Muslim	3	0	1	0	0	0	4(4.6%)
Other	2	1	0	0	0	1	4(4.6%)

## Thematic findings

We identified four main themes: illness interpretation (theme 1), social relationships and illness response (theme 2), medical pluralism (theme 3) and healthcare access (theme 4). Table 2 summarizes the thematic areas as well as the major findings for each area.

**Table 2.** Summary of emerging themes.

PASS Model Constructs	Themes	Summary of findings
Illness perception	Illness interpretation as naturally- or supernaturally- caused	Illness was divided into 2 major categories: supernatural etiology and biomedical etiology. The interpretation of symptoms and treatment determination is based on cultural beliefs.
Social values and stigma	Social relationships and illness response	The community and family are consulted when a child falls ill. They give advice on the best treatment course depending on their understanding of the illness. Fathers tend not to be included in the child healthcare decision making process.
Social pressure and support	Social support and pressure in healthcare seeking	Community and family members often offer advice to caregivers on appropriate treatment, caregivers usually oblige. In a show of support, caregivers are sometimes escorted to see traditional healers.
Access to care and resource seeking	Healthcare access	Financial difficulty was revealed to be a barrier to health care access for children. Though sick children were referred to higher level facilities there was reluctance to take them to the referral hospital or they did not have financial capability to heed the call to take a child to a referral facility.

### Illness interpretation

Most respondents reported that there are different kinds of illness, those caused by supernatural powers and those caused by biological factors. Respondents report symptoms appraisals based on illness interpretation which is arrived at from symptoms exhibited or events preceding the illness. Depending on the perceived etiology of the illness, the caregiver would seek appropriate care for the illness.

*“I’d say it depends on the sickness that you are seeking treatment for. Let’s say for children you’ll look at the type of sickness they have. It could be measles; most people always go for traditional remedies. You make the child sniff bhang [marijuana] and it [measles] goes away and if it is malaria you will either rush to Russia [Jaramogi Oginga Odinga Teaching and Referral Hospital] or District [Kisumu County Referral Hospital].”*
                    (Male, Kisumu FGD1)

Some respondents described how some illnesses require traditional medicine depending on what kind of illness it is, and if conventional medicine is sought instead, then dire consequences such as death may occur:

*“Our community has a disease called the small disease [locals refer to measles by a term that loosely translates as ‘the small disease’]. They believe that if a child who has contracted the disease is injected [as they would usually do in hospitals], then the child will not recover; they don’t recover. So they believe that they must be given ‘medicine of the pot’ [herbal medicine prepared by boiling in a pot].”*
                    (Female, Siaya FGD2)

According to the respondents, the community attributes illness to events. For example, an ill child may become sick because of something the mother did that’s considered a taboo by the community.

*“There are illnesses that can ail a child that is called ‘Chira’ [illness caused to punish wrongdoing]. The child can get it from the mother if she does something wrong[(taboo] then they would look for ‘manyasi’ [herbal concoction that remedies the effects of doing something against cultural norms]”*
                    (Male, Siaya FGD3)

Symptoms are interpreted into a diagnosis, and then the appropriate treatment is sought:

*“There is one called “okul bat”. This disease makes the child congested and the body becomes feverish. You just massage the child using Rob [a mentholated ointment] and OMO [handwashing powder] then he/she sweats a bit, sleeps and then they are cured.”*
                    
*(Male, Kisumu FGD1)*

Most of the respondent’s report that there is a common belief among community members who believe that any illness that does not fit a medical diagnosis is caused by witchcraft:

*“…I have an experience. My child was sick and when you looked at the child you could see that he was truly unwell. When I went to Russia I was told that there was no disease so when I came back I was told that it was witchcraft.”*
                    (Female, Kisumu FGD2)

### Role of social relationship on illness recognition and response

The respondents reported a communal approach to treatment of child illness: Whenever a child is ill, family, friends and neighbors are consulted about the illness. Due to longstanding cultural beliefs, community diagnosis is common and every so often appropriate course of action is derived. Mostly, traditional medicine is sought for cases believed to have a supernatural etiology:

*“When I gave birth to my first born, my child had symptoms such as fever and sweating. I took him to the hospital and when he was tested he was found not to be having malaria. When I came back with him I was not seeing any changes and a neighbor told me that “your child might have been flushed” [bewitched] and she took me to someone. When we went to that person he told me that my child had been bewitched even before I could say what had taken me there. There is something he did and he removed some things from the child and from there the child was alright.”*
                    (Female, Kisumu FGD1)

The respondent report that consultations among community members may result into switching from one medicine to the other depending on how the people consulted understand the illness thus causing confusion to the caregiver:

*“So you have two medicines, the traditional one and the one from the doctor. So when I go back home, you find you are being advised by the neighbor to first administer the traditional one. So you have two different medicines and when you give them to the child, they end up not working on the body of the child and you end up losing the child.”*
                    (Female, Kisumu FGD1)

A few respondents agree that treatment in their community is communal:

*“…The child was treated with herbal medicine because the people from Ugenya are knowledgeable in traditional medicine. They tried all traditional medicine on my child until my child became a zombie [non-responsive]. Now we were just waiting for him to die and be buried. My mum then asked me to come back to Kisumu and take the child to the hospital… my child was admitted and she took almost one month but when I was discharged the child had improved a lot. The child is alive to date...”*
                    (Female, FGD1 Kisumu)

However, a few respondents explained that in some cases the father can be left out in the decision making process of care seeking for the child:

*“…you may find that a woman has taken a child to hospital and she may be referred to Siaya [county referral hospital]. This matter it is only her who knows it in her heart…When she goes back home those drugs that she was given to use are what she will use. Instead of even telling the father [husband] that she was told to take the child to Siaya [referral], she will only try to give the child those drugs she had been given but she knows very well that she was told to go there [hospital she was referred to]*
                    (Male, Siaya FGD2)

### Social pressure and support in healthcare seeking

Caregivers report receiving advice from relatives and community members regarding the appropriate treatment for illnesses. Their advice typically reflects the practice of medical pluralism, where both biomedical or traditional treatment are used, depending on the interpretation of the illness. While the advice given may be perceived as social support, it can also manifest as social pressure where caregivers maybe compelled to follow the advice, even if it is not in line with their preference or judgement.

Respondents reported using either biomedicine or traditional medicine primarily due to advice from their family or community members. This advice to opt for traditional medicine stemmed from deeply ingrained health beliefs regarding various illnesses. It was uncommon for people outside the family to advise mothers on the best course of action. Most often, caregivers complied with these suggestions.

*“When I gave birth to my first born, my child had symptoms such as fever and sweating. I took him to the hospital and when he was tested he was found not to be having malaria. When I came back with him I was not seeing any changes and a neighbor told me that “your child might have been flushed” (bewitched) and she took me to someone. When we went to that person (witchdoctor) he told me that my child had been bewitched even before I could say what had taken me there. There is something he did and he removed some things from the child and from there the child was alright.”*
                    (Female, KII Kisumu)

When caregivers were living away from extended family, neighbors often advised caregivers on treatment options. This external influence further reinforced the reliance on traditional practices, contributing to the decisions made by caregivers in managing health issues. These pieces of advice can sometimes be confusing to caregivers.

*“So you have two medicines, the traditional one and the one from the doctor. So when I go back home, you find you are being advised by the neighbor to first administer the traditional one. So you have two different medicines and when you give them to the child, they end up not working on the body of the child and you end up losing the child.”*
                    (Female, KII Kisumu)

Respondents reveal that the advice can be fatal, as seen in the case of a caregiver in the quote below.

*…He was sick for a while; malaria was in his blood for long. He had gone with the mother to visit her paternal home when the child became sick but they thought it was ‘sihoho’ [folk illness]. They tried to treat it with local herbs but it was not ‘sihoho’. Eventually my child was brought home and died in the doctor’s hands at the dispensary here.*
                    (Male, Siaya FGD3)

The respondents reported that it is common for caregivers to alternate between biomedicine and traditional medicine depending on their own or family’s interpretation of the disease and severity of symptoms they see in their child through the communal support to make decisions, caregivers would start treatment at the hospital and switch to traditional medicine when biomedicine is ‘slow’ or illness is not improving:

* “There are some people who say that they go to hospital to seek treatment but they find that the treatment isn't helpful. So they or their family may have alternative thoughts, then they decide to go to faith healers or they go to traditional herbalists.”*
                    (Male, KII Kisumu)

### Healthcare access

The respondents explained that a patient may be unable to seek prescribed care because they cannot afford services at the health facilities from the formal healthcare system. Although they started seeking healthcare services at the health facility, they may choose to go to traditional healers who are perceived to be more affordable:

* “Maybe when a person comes to the hospital and you refer her/him and then he/she thinks that she will not be able to afford to pay at the county referral, he will choose to stay and seek herbal treatment.”*
                    (Female, FGD3 Siaya)

A few respondents believe that health services at the government facility is free of charge:

*...the government health centers that we have are free then apart from being free we can’t afford the private ones because of lack of income. That’s what makes most of us not go to the private ones.*
                    (Female, Siaya FGD2)

During the discussions, the respondents state that frequent strikes in government health facilities and healthcare workers´ frequent strikes has made the community look for health care elsewhere. When healthcare workers in public health facilities are on strike, caregivers have to seek alternative healthcare seeking:

*“Manyatta community, nowadays they don’t trust the government hospital because of strikes every now and then, Strike! Strike! Strike! So they prefer these private hospitals and these pharmacies. Some just go and buy drugs from the pharmacies, yes.”*
                    (Female, SSI Kisumu)

## Discussion

This study revealed that caregivers’ interpretation of childhood illness is reflected in the treatment-seeking behavior for child illness in western Kenyan communities. Illnesses were believed to have two broad etiologies, supernatural and biological. Interpretation is made based on signs and symptoms of a particular illness and presumed cause of illness ^23^. Often illnesses with supernatural etiology such as those that follow breaching a taboo or caused by witchcraft are first treated using traditional medicine, a finding which is congruent with previous studies ^24– 26^. Measles was one such disease, with severe consequences to be expected if biomedicine was chosen instead. Similarly, in Tanzania, *degedege*, a folk illness with symptoms of malaria, was given mystical etiology and treatment with traditional medicine prioritized to avoid death ^23, 27^. Beliefs about the cause of the illness results in customs and practices that can adversely affect illness outcome ^28^. Our findings report that malaria was recognized as a biologically-caused illness, and this shows caregivers’ awareness of malaria symptoms. These findings are similar to other studies which report that malaria symptoms were easily recognized and treated at the hospital ^29, 30^.

The respondents report a communal approach to finding treatment for childhood illness where a caregiver with a sick child would be advised on appropriate care for the child. PASS framework posit that health-seeking behavior is founded on social values whereby a particular behavior is expected ^12^. Seeking and receiving advice was socially expected from caregivers of young children in this context. Therefore, social relationship is important in illness recognition and response and is derived from social values. Consultation with neighbors, mother in law and spouse is common in our setting, similar to other studies findings ^6, 17^. In urban areas, where caregivers lived far from their extended family, neighbors stepped in to provide caregivers with care-seeking advice. This was noted to contribute to delays in seeking healthcare ^6, 26, 30^. Some studies have established the importance of fathers’ financial support and participation in childhood illness ^31– 33^, however, our findings show that fathers were not involved in a child’s treatment because mothers concealed doctor’s advice about child’s treatment. Concealing doctors' advice may hinder father’s involvement or participation in decision making and this may deny a child clinical management that they need to restore health. Therefore, there is a need to understand why mothers are not involving fathers in health seeking for children.

Consistent with previous reports, medical pluralism was common among caregivers ^34, 35^. The treatment course varies with some starting treatment at the health facility then switching to a traditional medicine when the illness gets worse. These decisions are often consultative as is socially expected which can pressurize caregivers to oblige to advice on appropriate treatment. Price’s study conducted in South Africa among caregivers seeking care during a fatal childhood illness report that traditional medicine was used as a last resort when caregivers were feeling desperate or when the illness gets worse ^36^. Hooft, *et al.* also reported that most caregivers sequentially seek multiple healthcare providers and treatment modalities until there is a perceived benefit ^17^. Our findings reveal gaps in communication between the medical practitioner and caregiver, as the medical practitioner fails to inform caregiver of treatment and progress with the caregiver, the caregiver isn’t aware of what to expect. This shows a need for counseling for caregivers with critically ill children who may get desperate when they don’t notice immediate improvement. Our findings also reveal that treatment with traditional medicine first caused delays in definitive care leading to severe illness and death. Previous research has also documented the importance of prompt clinical management to avert severe morbidity ^34^. These findings show a nonlinear health care seeking pathway as described by the PASS model where health care seeking behavior is made and reevaluated depending on the patient’s response to treatment ^10, 15^.

The 2030 Sustainable Development Goals emphasize having all people receive quality care without financial hardship ^3^. Community members find healthcare unaffordable in private facilities during public health worker’s strikes. During these periods, caregivers consult local pharmacies which they perceive to be affordable, a finding consistent with a study conducted in Kilifi county in Eastern Kenya ^37^. Referral to higher levels of care is an important component of child survival, but caregivers report not being able to afford to take their children to referral facilities. This leads them to seek alternative treatment which are perceived to be more affordable and still effective. This shows a gap in the implementation of the integrated management of childhood illness (IMCI) strategy which aims at strengthening referral pathways to improve health outcomes ^38^. The decision not to take a child to the referred facility is still not clearly understood given the fact that Free Health Care Initiative (FHCI) and Universal Health care was instituted to protect vulnerable populations from catastrophic expenditures and to promote equity in health care provision ^37^. The full cost to a family of a very sick child’s care needs to be better understood in order to improve healthcare access in our setting, particularly as other studies in the region have also reported that poverty can be a major deterrent to ‘appropriate’ health care seeking behavior.

This study has four main limitations. First, we asked caregivers about hypothetical situations, and did not describe the caregivers’ actual behavior and rationale for actions. Second, we did not explore perceptions about health services, which can influence health seeking behavior. Finally, it is possible that participants expressed what they perceived to be appropriate or socially desirable responses.

## Conclusion

Health-seeking decisions are driven by both intrinsic and extrinsic factors, and understanding community drivers to health-seeking behavior is important in formulating policies and interventions that improve health outcomes. Our findings indicate that a strong health education program at community level could improve caregivers’ ability to interpret signs and symptoms of common childhood diseases, understand danger signs that require immediate clinical intervention, and involve fathers in decision-making around healthcare for their children. We recommend involving caregivers in the development of educational program interventions ensures that the messages are tailored to the audience’s needs. This collaboration helps create more effective and relevant health promotion strategies.

## Data Availability

The data provided cannot be made available due to ethical constraints from the Kenya Medical Research Institute- Scientific Ethics Review Unit (KEMRI-SERU), as participants have not provided consent for their data to be stored in a public repository. KEMRI-SERU state that anonymized data can be shared under specific requests via the corresponding author.
