# Supplementary material for: Health care-seeking behavior for childhood illnesses in western Kenya: Qualitative findings from the Child Health and Mortality Prevention Surveillance (CHAMPS) Study
Source: Gates Open Res. 2024 Sep 27;8:31. Originally published 2024 Apr 24. [Version 3] doi: 10.12688/gatesopenres.14866.3 (PMC11413273; doi:10.12688/gatesopenres.14866.3)
Supplement: Peer review history

# Reviewer report: Reviewer response for version 3

**DOI:** 10.21956/gatesopenres.17687.r38758

**Published:** 2024-12-20

**Authors:** Md Aminul Haque

**Affiliations:** 1 Department of Population Sciences, University of Dhaka, Dhaka, Bangladesh

Dear Authors, thank you very much for the nice work done. 

 Would it be possible to give a figure or flow chart in the manuscript using those aspects ( If it is not possible for the authors to draw the linkup/flowchart then also I approve the manuscript) : 

 1. A figure with the steps of the path that respondents follow when seeking care may be added.

 2. How was illness interpretation developed and influenced? and influenced Health Care seeking

 3. How does access to availability and accessibility influence Health Care seeking? 

 4. How are interpretations of illness, acceptability/availability linked to healthcare decision-making? and

 5. Finally, how are these linked to medical pluralism?

Is the work clearly and accurately presented and does it cite the current literature?

Yes

If applicable, is the statistical analysis and its interpretation appropriate?

Not applicable

Are all the source data underlying the results available to ensure full reproducibility?

Yes

Is the study design appropriate and is the work technically sound?

Yes

Are the conclusions drawn adequately supported by the results?

Yes

Are sufficient details of methods and analysis provided to allow replication by others?

Yes

Reviewer Expertise:

Both Quantitative and Qualitative research, Population Health, Aging, Women's Empowerment, maternal, newborn and child health

I confirm that I have read this submission and believe that I have an appropriate level of expertise to confirm that it is of an acceptable scientific standard.

# Reviewer report: Reviewer response for version 3

**DOI:** 10.21956/gatesopenres.17687.r38168

**Published:** 2024-11-06

**Authors:** Alemayehu Amberbir

**Affiliations:** 1 University of Global Health Equity, Kigali, Rwanda

No further comments.

Is the work clearly and accurately presented and does it cite the current literature?

Yes

If applicable, is the statistical analysis and its interpretation appropriate?

Yes

Are all the source data underlying the results available to ensure full reproducibility?

Partly

Is the study design appropriate and is the work technically sound?

Yes

Are the conclusions drawn adequately supported by the results?

Yes

Are sufficient details of methods and analysis provided to allow replication by others?

Partly

Reviewer Expertise:

Global health epidemiologist, health system research in Africa, implementation research, non-communicable diseases

I confirm that I have read this submission and believe that I have an appropriate level of expertise to confirm that it is of an acceptable scientific standard.

# Reviewer report: Reviewer response for version 3

**DOI:** 10.21956/gatesopenres.17687.r38169

**Published:** 2024-10-18

**Authors:** Alyssa Sharkey

**Affiliations:** 1 Princeton University, Princeton, New Jersey, USA

I have read through the authors’ responses to my earlier queries and am satisfied with their responses. I approve this article.

Is the work clearly and accurately presented and does it cite the current literature?

Yes

If applicable, is the statistical analysis and its interpretation appropriate?

Not applicable

Are all the source data underlying the results available to ensure full reproducibility?

No

Is the study design appropriate and is the work technically sound?

Yes

Are the conclusions drawn adequately supported by the results?

Partly

Are sufficient details of methods and analysis provided to allow replication by others?

Partly

Reviewer Expertise:

Qualitative research, global health, maternal, newborn and child health

I confirm that I have read this submission and believe that I have an appropriate level of expertise to confirm that it is of an acceptable scientific standard.

# Reviewer report: Reviewer response for version 2

**DOI:** 10.21956/gatesopenres.17662.r37756

**Published:** 2024-09-19

**Authors:** Alemayehu Amberbir

**Affiliations:** 1 University of Global Health Equity, Kigali, Rwanda

This study qualitatively investigates the healthcare-seeking behaviors of both the community and providers regarding childhood illnesses in Kenya. It offers valuable contextual insights into the factors influencing these behaviors and suggests potential interventions aimed at reducing child mortality in these settings.

 However, a significant limitation is that the research was conducted seven years ago, raising concerns about the relevance of the findings in today's context. This time gap makes it challenging for general readers to assess how applicable the results are in the current healthcare landscape. The background section should be updated to reflect the latest developments in this area.

 Additionally, given that the study was conducted prior to the COVID-19 pandemic, it is worth considering whether any of the findings may have changed in light of the pandemic's impact on healthcare-seeking behaviors.

 Furthermore, the application of the PASS theoretical framework is not clearly articulated. It is important to clarify whether this framework was applied to both providers and caregivers in the analysis.

 Minor Comment:

1. The Methods section contains a repeated ethics approval statement on pages 4 and 5. Please review and amend accordingly.

Is the work clearly and accurately presented and does it cite the current literature?

Yes

If applicable, is the statistical analysis and its interpretation appropriate?

Yes

Are all the source data underlying the results available to ensure full reproducibility?

Partly

Is the study design appropriate and is the work technically sound?

Yes

Are the conclusions drawn adequately supported by the results?

Yes

Are sufficient details of methods and analysis provided to allow replication by others?

Partly

Reviewer Expertise:

Global health epidemiologist, health system research in Africa, implementation research, non-communicable diseases

I confirm that I have read this submission and believe that I have an appropriate level of expertise to confirm that it is of an acceptable scientific standard, however I have significant reservations, as outlined above.

# Response

**Published:** 2024-09-25

**Authors:** Sarah Ngere

**Affiliations:** Socio-behavioral department, Kenya Medical Research Institute, Kisumu, Kisumu, Kenya

The authors would like to thank the reviewer for reviewing and providing comments. We have addressed all the comments below. 

**Reviewer Comment:**

 This study qualitatively investigates the healthcare-seeking behaviors of both the community and providers regarding childhood illnesses in Kenya. It offers valuable contextual insights into the factors influencing these behaviors and suggests potential interventions aimed at reducing child mortality in these settings.

 However, a significant limitation is that the research was conducted seven years ago, raising concerns about the relevance of the findings in today's context. This time gap makes it challenging for general readers to assess how applicable the results are in the current healthcare landscape. The background section should be updated to reflect the latest developments in this area.

**Author Response: **Thank you for the comment. The authors appreciate the concern of the time taken to publish these results. The authors included more recent papers in the areas in the introduction section.

**Reviewer Comment:**

 Additionally, given that the study was conducted prior to the COVID-19 pandemic, it is worth considering whether any of the findings may have changed in light of the pandemic's impact on healthcare-seeking behaviors.

**Author Response: **Thank you for your comment. The authors acknowledge the difference in pre and post COVID-19 pandemic in regards to care-seeking. However, the study was conducted pre-COVID and therefore we cannot provide any findings at this time. We will take this as a recommendation.

**Reviewer Comment:**

 Furthermore, the application of the PASS theoretical framework is not clearly articulated. It is important to clarify whether this framework was applied to both providers and caregivers in the analysis.

**Author Response: **Thank you for the comment. The study had a wide range of participants. We used the PASS framework to understand motivations to their health seeking behavior. The constructs of the PASS frame-work directly address individual motivations which in this case are the caregivers and not the healthcare workers.

**Reviewer Comment:**

 Minor Comment:

 The Methods section contains a repeated ethics approval statement on pages 4 and 5. Please review and amend accordingly.

**Author Response: **Thank you for the comment. One ethical approval statement has been removed.

# Reviewer report: Reviewer response for version 2

**DOI:** 10.21956/gatesopenres.17662.r37626

**Published:** 2024-08-19

**Authors:** Alyssa Sharkey

**Affiliations:** 1 Princeton University, Princeton, New Jersey, USA

Response to reviewer

 The authors would like to thank the reviewer for the comments and insights provided during the review.

 We acknowledge that education and religion can influence perceptions. However, we found no difference in perception among participants across various socio-demographic characteristics. This is likely because the participants' perceptions and responses were more influenced by their shared culture, which was similar among all participants.

**New response:** I might need to clarify my concern regarding this point which was that it would is not possible to determine whether or not there is a difference in perceptions among participants by the socio-demographic characteristics of education and religion because your sample has very few individuals from these groups that I mentioned. So this should be included as a limitation of the study unless there are very few individuals of Muslim or “Other” religions among populations in Western Kenya, and if almost all caregivers have an education. That seems unlikely to me, so I am suggesting that this may be a limitation.

**The constructs within the PASS model need more clarification. It is not immediately clear what the difference is between ‘social values and stigma’ and ‘social pressure and support’ – specifically, why wouldn’t medical pluralism also represent social values?**

 Thank you for your observation. The authors acknowledge that the constructs within the PASS model was not immediately clear to readers. Therefore, the section has been rewritten. Medical pluralism as a title and results was not brought out clearly to showcase social pressure and support which the authors initially implied. The authors have reworked this section to bring out clearly the construct social pressure and support. Under the new theme, social support, the results show that caregivers receive support through advice from community and friends on the best course of action when a child is ill. The title and results have been reworked for clarity.

 Grammar and typo: Thank you for the comment. We have checked the whole document for grammatical errors and typos.

**New response:** The Theoretical Framework section of the paper still only lists the 4 constructs without clarifying the differences. I could not find where these are described and differentiated within the paper.

Conclusion

**The conclusion places too much emphasis on the need to better educate local caregivers, when the study clearly highlights the range of barriers that need to be addressed. The conclusion should also emphasize that any solutions developed to address the identified barriers should incorporate the inputs of the local community (e.g., to ensure a human-centered design approach).** 

 Thank you for the comment. We have included a recommendation to involve caregivers in the process of message/intervention development.

**New response:** Recommendations should be made relating to all the themes (including the financial barriers theme). While it looks like you have included this within the Discussion, this should also be added to the Conclusions.

**New reviewer comments**

**Background: “**Using the constructs of the Partners for Applied Social Sciences (PASS) model, we explored how cultural and social factors drove health care decision making.” I would say you also explore economic factors (i.e., in the healthcare access theme)

**Results**: The four stated constructs of the PASS framework are i) illness perception; ii) social values and stigma; iii) social pressure and support; iv) access to care and resource seeking. However, now the four themes listed within the Results section are: illness interpretation, social relationships and illness response, medical pluralism and healthcare access. 

 Given how different the emerging themes are from the PASS constructs (particularly themes 2 and 3), it is not clear why the PASS is still included as the theoretical framework. At least there should be a discussion (in the Discussion section) about how the two new themes arose in spite of the guiding framework.

 The first two examples included under the heading “Role of social relationship on illness recognition and response” seem to overlap significantly with the examples included under “Illness interpretation.” For example, compare the text of the last quote under “Illness interpretation” (which ends with “…when I came back I was told that it was witchcraft”) with these first two quotes (which also indicate that someone else telling the caregiver what the illness cause was). It is still not completely clear how the authors are trying to distinguish these two themes.

 Similarly, there is overlap with what is still included under the heading “Social support in healthcare seeking.” In fact, the quote that begins, “When I gave birth to my first born….” has been used in both sections. This is also true of the quote that begins, ““So you have two medicines…”

 This makes the Results section confusing. Perhaps these two themes need to be combined, and then all duplicate quotes should be dropped.

**Discussion**: I think you can drop the following sentence: “First, our findings are contextual and may not be generalizable to the wider country context.” Generalizability is not an aim of qualitative methods (as opposed to in-depth, contextually specific analysis) and therefore should not be considered a limitation.

Is the work clearly and accurately presented and does it cite the current literature?

Yes

If applicable, is the statistical analysis and its interpretation appropriate?

Not applicable

Are all the source data underlying the results available to ensure full reproducibility?

No

Is the study design appropriate and is the work technically sound?

Yes

Are the conclusions drawn adequately supported by the results?

Partly

Are sufficient details of methods and analysis provided to allow replication by others?

Partly

Reviewer Expertise:

Qualitative research, global health, maternal, newborn and child health

I confirm that I have read this submission and believe that I have an appropriate level of expertise to confirm that it is of an acceptable scientific standard, however I have significant reservations, as outlined above.

# Response

**Published:** 2024-09-25

**Authors:** Sarah Ngere

**Affiliations:** Socio-behavioral department, Kenya Medical Research Institute, Kisumu, Kisumu, Kenya

**Response to comments**

 The authors would like to thank you for reviewing the manuscript and providing comments which the authors have found very useful. We have carefully addressed all the comments below.

**New reviewer comments**

**Background: “Using the constructs of the Partners for Applied Social Sciences (PASS) model, we explored how cultural and social factors drove health care decision making.” I would say you also explore economic factors (i.e., in the healthcare access theme).**

**Author Response: **Thank you the comment. We have added economic factors to the statement.

**Reviewer Comments:**

**Results:** The four stated constructs of the PASS framework are i) illness perception; ii) social values and stigma; iii) social pressure and support; iv) access to care and resource seeking. However, now the four themes listed within the Results section are: illness interpretation, social relationships and illness response, medical pluralism and healthcare access.

 Given how different the emerging themes are from the PASS constructs (particularly themes 2 and 3), it is not clear why the PASS is still included as the theoretical framework. At least there should be a discussion (in the Discussion section) about how the two new themes arose in spite of the guiding framework.

**Author Response: **Thank you for the comment. The authors have included an explanation of theme 2; the role of social relationships and illness response in the discussion section on paragraph 2. We have maintained the original PASS framework theme; social pressure and support and provided clarity in the result section.

**Reviewer Comments:**

 The first two examples included under the heading “Role of social relationship on illness recognition and response” seem to overlap significantly with the examples included under “Illness interpretation.” For example, compare the text of the last quote under “Illness interpretation” (which ends with “…when I came back I was told that it was witchcraft”) with these first two quotes (which also indicate that someone else telling the caregiver what the illness cause was). It is still not completely clear how the authors are trying to distinguish these two themes.

 Similarly, there is overlap with what is still included under the heading “Social support in healthcare seeking.” In fact, the quote that begins, “When I gave birth to my first born….” has been used in both sections. This is also true of the quote that begins, ““So you have two medicines…”

**Author Response:** Thank you for the comment. The authors would like to keep the repeated quote in the 2 different themes because it portrays both the role of social relationship on illness recognition and response and social pressure and support in healthcare seeking which are explained below.

**Reviewer Comments:**

 This makes the Results section confusing. Perhaps these two themes need to be combined, and then all duplicate quotes should be dropped.

**Author Response:** Thank you for the observation and comment. The issues you have mentioned here are pertinent and the authors have explained the difference between the 3 themes below - 

**Illness interpretation:**  According to our findings illness was categorized into having biomedical and supernatural etiology. Authors explain how the different ways of interpreting illness influence health seeking behavior.  Illness interpretation therefore influences the kind of treatment a caregiver would give her/his child for the particular illness.

**Role of social relationship on illness recognition and response:** The findings indicate how social relationship influence health seeking behavior by recognizing the illness. Our data reveal that cultural beliefs held by others would greatly influence the course of action for caregiving.

**Social pressure and support in healthcare seeking: **The authors agree that there was an overlap between theme number 2 and 3 which was confusing and therefore wish to give the 3 ^rd^ theme more clarity. The PASS framework 3 ^rd^ construct is social pressure and social support which the authors would want to maintain. We have therefore made theme clearer by explaining the overlap between social support and pressure in the context of health seeking. Community members may feel pressurized to take up support during sickness which can be offered as advise or practical care-seeking (taking them for treatment) depending on the illness.

**Reviewer Comments:**

**Discussion: I think you can drop the following sentence: “First, our findings are contextual and may not be generalizable to the wider country context.” Generalizability is not an aim of qualitative methods (as opposed to in-depth, contextually specific analysis) and therefore should not be considered a limitation.**

**Author Response: **Thank you for the suggestion the statement has been dropped in the revised version.

# Reviewer report: Reviewer response for version 2

**DOI:** 10.21956/gatesopenres.17662.r37635

**Published:** 2024-08-16

**Authors:** Oluseye Ademola Okunola

**Affiliations:** 1 Obafemi Awolowo University, Ife, Osun, Nigeria; 2 Sociology and Anthropology / Medical and Health Services, Obafemi Awolowo University, Ife, Osun, Nigeria

The study offers useful insights into caregivers' experiences and perceptions, emphasizing the intricacies of healthcare-seeking behavior.

 The application of qualitative approaches generates rich, contextualized data.

 The study's focus on western Kenya fills a significant gap in understanding healthcare-seeking behavior in the region. However, the study's small sample size and geographic coverage may restrict generalization, and the lack of quantitative data and healthcare practitioner opinions may result in an incomplete picture.

 In conclusion, this article provides important qualitative insights into healthcare-seeking behavior for pediatric diseases in western Kenya. Despite several limitations, the study's findings have significant implications for improving healthcare access and outcomes in this region. Future study could build on these findings, combining quantitative data and viewpoints from healthcare providers to produce a more comprehensive understanding.

Is the work clearly and accurately presented and does it cite the current literature?

Yes

If applicable, is the statistical analysis and its interpretation appropriate?

Yes

Are all the source data underlying the results available to ensure full reproducibility?

Yes

Is the study design appropriate and is the work technically sound?

Yes

Are the conclusions drawn adequately supported by the results?

Yes

Are sufficient details of methods and analysis provided to allow replication by others?

Partly

Reviewer Expertise:

My Research areas include, health systems services and health behavior research.

I confirm that I have read this submission and believe that I have an appropriate level of expertise to confirm that it is of an acceptable scientific standard.

# Response

**Published:** 2024-09-25

**Authors:** Sarah Ngere

**Affiliations:** Socio-behavioral department, Kenya Medical Research Institute, Kisumu, Kisumu, Kenya

The authors would like to thank you for reviewing the article and providing feedback.

# Reviewer report: Reviewer response for version 1

**DOI:** 10.21956/gatesopenres.16194.r37169

**Published:** 2024-07-19

**Authors:** Alyssa Sharkey

**Affiliations:** 1 Princeton University, Princeton, New Jersey, USA

This study focuses on an important topic that has been widely studied but also requires qualitative analysis within local settings to understand the specific barriers and solutions that might contribute to improved care-seeking among families with young children. This study focuses on Western Kenya which is an important and disadvantaged part of the country.

 It is unfortunate that the data were collected over 6 years ago and are only being published now as the key factors influencing care-seeking behaviors may have changed since that time. However, the study likely has yielded many findings that continue to have relevance today.

 I also note that only one caregiver without an education was included within the sample. It is possible that this sub-group might face different or additional to barriers to care-seeking so was perhaps not adequately represented within the study. Similarly, there are few respondents included in Table 2 who stated that they were Muslim or “Other” religions – this may be another limitation of the sample.

 The constructs within the PASS model need more clarification. It is not immediately clear what the difference is between ‘social values and stigma’ and ‘social pressure and support’ – specifically, why wouldn’t medical pluralism also represent social values?

Results

 In table 2, the text under “summary of findings” for ‘social values and stigma’ has typos:

 “The community and family are consulted when a child falls ill. They give advice on the best treatment course depending on their understanding of the illness. Fathers tend not to be included in the child healthcare decision making process.”

 I was surprised that previous experiences with health services were not found to be a determinant of care-seeking although it seems this is not even considered within the PASS framework so was not explored. This is a limitation and might explain not just medical pluralism but rejection of conventional health services.

Discussion

 Were there important differences between the urban and rural areas that could be reported?

Conclusion

 The conclusion places too much emphasis on the need to better educate local caregivers, when the study clearly highlights the range of barriers that need to be addressed. The conclusion should also emphasize that any solutions developed to address the identified barriers should incorporate the inputs of the local community (e.g., to ensure a human-centered design approach).

Overall

 There are a few areas in the paper where grammatical correction needed. Please see the attached PDF where I have marked some of these. I have also included some specific comments within the PDF.

Is the work clearly and accurately presented and does it cite the current literature?

Yes

If applicable, is the statistical analysis and its interpretation appropriate?

Not applicable

Are all the source data underlying the results available to ensure full reproducibility?

No

Is the study design appropriate and is the work technically sound?

Yes

Are the conclusions drawn adequately supported by the results?

Partly

Are sufficient details of methods and analysis provided to allow replication by others?

Partly

Reviewer Expertise:

Qualitative research, global health, maternal, newborn and child health

I confirm that I have read this submission and believe that I have an appropriate level of expertise to confirm that it is of an acceptable scientific standard, however I have significant reservations, as outlined above.

# Response

**Published:** 2024-08-02

**Authors:** Sarah Ngere

**Affiliations:** Socio-behavioral department, Kenya Medical Research Institute, Kisumu, Kisumu, Kenya

Response to reviewer

 The authors would like to thank the reviewer for the comments and insights provided during the review.

 We acknowledge that education and religion can influence perceptions. However, we found no difference in perception among participants across various socio-demographic characteristics. This is likely because the participants' perceptions and responses were more influenced by their shared culture, which was similar among all participants.

**The constructs within the PASS model need more clarification. It is not immediately clear what the difference is between ‘social values and stigma’ and ‘social pressure and support’ – specifically, why wouldn’t medical pluralism also represent social values?**

 Thank you for your observation. The authors acknowledge that the constructs within the PASS model was not immediately clear to readers. Therefore, the section has been rewritten. Medical pluralism as a title and results was not brought out clearly to showcase social pressure and support which the authors initially implied. The authors have reworked this section to bring out clearly the construct social pressure and support. Under the new theme, social support, the results show that caregivers receive support through advice from community and friends on the best course of action when a child is ill. The title and results have been reworked for clarity.

 Grammar and typo: Thank you for the comment. We have checked the whole document for grammatical errors and typos.

**I was surprised that previous experiences with health services were not found to be a determinant of care-seeking although it seems this is not even considered within the PASS framework so was not explored. This is a limitation and might explain not just medical pluralism but rejection of conventional health services.**

 Thank you for the observation. The authors agree that previous experiences with health services were not explored. We have included the limitation in the limitation section.

Discussion

**Were there important differences between the urban and rural areas that could be reported?**

 Thank you for the comment. Consultation with family within the rural area was observed however, in urban areas caregivers received care-seeking report receiving support from neighbors. No other difference was observed.

Conclusion

**The conclusion places too much emphasis on the need to better educate local caregivers, when the study clearly highlights the range of barriers that need to be addressed. The conclusion should also emphasize that any solutions developed to address the identified barriers should incorporate the inputs of the local community (e.g., to ensure a human-centered design approach).** 

 Thank you for the comment. We have included a recommendation to involve caregivers in the process of message/intervention development.

Overall

**There are a few areas in the paper where grammatical correction needed. Please see the attached PDF where I have marked some of these. I have also included some specific comments within the PDF**.

 We were unable to locate the PDF however, we have gone through the entire document and corrected all grammatical errors.
